# Inquiry into the Temperature Changes of Rock Massif Used in Energy Production in Relation to Season

**DOI:** 10.3390/s21217027

**Published:** 2021-10-23

**Authors:** Martin Klempa, Jan Latal, Barbora Grafova, Michal Matloch Porzer, Mojmir Vrtek, Antonin Kunz, Petr Siska

**Affiliations:** 1 Department of Geological Engineering, Faculty of Mining and Geology, VSB–Technical University of Ostrava, 17. listopadu 2172/15, 708 00 Ostrava Poruba, Czech Republic; michal.matloch.porzer@vsb.cz (M.M.P.); antonin.kunz@vsb.cz (A.K.); 2 Department of Telecommunications, Faculty of Electrical Engineering and Computer Science, VSB–Technical University of Ostrava, 17. listopadu 2172/15, 708 00 Ostrava Poruba, Czech Republic; petr.siska@vsb.cz; 3 Department of Hydrodynamics and Hydraulic Equipment, Faculty of Mechanical Engineering, VSB–Technical University of Ostrava, 17. listopadu 2172/15, 708 00 Ostrava Poruba, Czech Republic; barbora.grafova@vsb.cz; 4 Department of Power Engineering, Faculty of Mechanical Engineering, VSB–Technical University of Ostrava, 17. listopadu 2172/15, 708 00 Ostrava Poruba, Czech Republic; mojmir.vrtek@vsb.cz

**Keywords:** borehole heat exchanger, ground temperature field, ground thermal conductivity, ground source heat pump, Raman-OTDR (DTS), temperature measurement

## Abstract

This research was undertaken to perform and evaluate the temperature measurement in the ground utilized as an energy source with the goal to determine whether significant temperature variations occur in the subsurface during the heating season. The research infrastructure situated on our University campus was used to assess any variations. The observations were made at the so called “Small Research Polygon” that consists of 8 monitoring boreholes (Borehole Heat Exchangers) situated around a borehole used as an energy source. During the heating season, a series of monthly measurements are made in the monitoring boreholes using a distributed temperature system (DTS). Raman back-scattered light is analysed using Optical Frequency Time Domain Reflectometry (OTDR). Our results indicate that no noticeable changes in temperature occur during the heating season. We have observed an influence of long-term variations of the atmospheric conditions up to the depth of a conventional BHE (≈100 m). The resulting uncertainty in related design input parameters (ground thermal conductivity) was evaluated by using a heat production simulation. Production data during one heating season at our research facilities were evaluated against the design of the system. It is possible to construct smaller geothermal installations with appropriate BHE design that will have a minimal impact on the temperature of the surrounding rock mass and the system performance.

## 1. Introduction

Borehole heat exchangers (BHEs) in ground source heat pump (GSHP) systems represent a significant potential for economically efficient heat gain or heat storage for objects of most types and sizes, particularly in buildings (or industrial processes) in which excess heat is produced episodically or periodically and there is no use for it at the time. Here, BHEs can serve as BTES (Borehole Thermal Energy Storage) systems [1].

Performance optimization of borehole heat exchangers is one of the key steps in their design and can significantly reduce the installation costs and risks that, in a long-term view, could thwart the investment due to the “overload” of the rock mass and its potential subsequent “freeze”. The aim of these methods is not only to evaluate and describe the mutual influence of the built (spatially close) systems, but also to enable the long-term development of temperatures in a rock massif [2]. Naturally, performance optimization approaches must be appropriate to the size of the installation, as advanced numerical methods are time and cost consuming.

Heat pumps, in the context of renewable energy sources for heating, production of hot water and cooling, play an important role in pursuing the Directive of the European Parliament and of the Council on the energy performance of buildings as a strategic commitment of all EU member countries. The number of ground source heat pump installations is steadily growing in Europe. The installation growth rate in the shallow geothermal sector is declining; a capacity of more than 20 GWth was achieved at the end of 2015, distributed in over more than 1.7 million installations in Europe [3].

Thermal energy, harnessed from heat pumps, reduces the overall fossil fuel consumption, which is aligned with the European Commission to reduce greenhouse gas emissions, emissions that are negatively influencing the climate, not only regionally, but also globally. For example, in the Czech Republic, the utilization of GSHPs (i.e., primary utilizing low temperatures of the bedrock) has saved our country approximately 25,000 tonnes of energy (toe) in fossil fuels usage [4].

The GSHP installations can be divided according to the connection of technology used in the systems, (1) the GSHP is only for heating, (2) GSHP installations are used for both heating and cooling by reversing the pump [5,6,7].

Among the most common GSHP with heat pumps installed in Central European climatic conditions are geothermal systems with boreholes in the bedrock that circulate heated fluid through a closed-loop, commonly known as collectors (i.e., GSHP; closed-loop system earth/water). These systems are suitable for all sizes of installations whose power and SCOP${}_{net}$ are not significantly influenced by seasonal climatic effects.

GSHP installations can be divided e.g., according to the desired heat output, or, more precisely, according to the desired amount of thermal energy either obtained from the surroundings or spent/accumulated into the surroundings over a period of time. In the literature, GSHP installations are usually divided as follows (see in Table 1):

The object of this research is to determine the extent of influence of thermal energy production during heating on the temperature field in the ground surrounding a system of BHEs (referred to as a polygon of BHEs). The polygon includes operational boreholes produced with the use of heat pumps) primarily used during the heating season (September–May). To achieve these research goals, VSB–Technical University Ostrava operates two research geothermal stations on its campus where long-term monitoring of subsurface temperature is conducted to detect changes in temperature profile of the surrounding rock mass. A distributed temperature sensing system (DTS) that analyses Raman back-scattered light using Optical Frequency Time Domain Reflectometry (OTDR) has proven to be the best tool for monitoring, and we have used the technology for many years to measure temperature changes in the bedrock [8,9,10,11].

Most of the research work in the past focused on the creation of very promising prediction models of GSHP systems’ behaviour, such as in [12,13], which makes it possible to understand the thermal impact of thermal energy production and various hydrogeological and flow conditions.

The optical measuring system Raman-OTDR was used for observing the heat changes inside the energetically utilized ground. This system for continual measuring of temperature changes has proved successful in e.g., hydrogeology, oil industry and building industries [9,14,15].

In the article [16] authors use of DTS systems for monitoring temperature gradients in hydrogeological wells. Another typical installation of DTS systems for hydrogeologic observations of temperature changes is studied in [17] where it has been found that DTS systems allow efficient temperature measurements in deep wells along their entire length as opposed to conventional electrical sensors located in wells in discrete points. The use of DTS systems for monitoring changes in time and space in oceanography was achieved by [18] where they measured the skin effect for three freshwater bodies at three selected locations (Netherlands, Israel and Ghana). A very detailed analysis of the use of DTS systems in a large number of sectors is elaborated in [19].

Many measurements employing DTS systems warranted its suitability for deployment in the cases of assessing the thermal behaviour of rock environment. In the [20,21] dealt with the heat transmission between the individual rock layers of a massif using this measuring system. Further results on the behaviour of individual layers of the massive rock were made in the [22], where they found that conducting a Thermal Response Test with stratified optical fibres in the space can be used to recognize stratification of soil properties along the depth of the borehole In another publication, [23] deals with A very important issue associated with geothermal boreholes, i.e., the passage of moisture or water through the massive rock along the borehole in temperature responses.

Initial testing of long-term temperature measurement using DTS systems was carried out in the area of Campi Flegrei caldera which includes a part of the large city of Naples (Italy). 500 m deep wells with high temperatures of about 100 ${}^{\circ }$C were measured in an area with volcanic activity [24]. The installation for 8 days and temperature measurement using the DTS system were carried out in geothermal wells in the production state. Based on the measured data it was possible to determine cold or anomalously warm zones around the used geothermal wells [15].

Our goal is to assess the influence of the thermal energy production on the ground temperature field. Initial qualitative evaluation of BHE temperature logs will allow us to observe any temperature changes induced by the influence of GSHP operation. The intensity of such an influence is dependent on the heat load inflicted upon the ground by the GSHP installation. Resulting decrease (or increase) in ground temperature develops in a shape of “radial” depression around a BHE. Shape and extent of the depression depends mainly on the coefficient of thermal conductivity of the ground. After our initial observation we will therefore evaluate how our experimental installation performs relative to predicted performance during its design. We will attempt to discuss all usual methods of obtaining ground parameters and their influence on the performance of our experimental installation. This is an attempt to touch the influence of uncertainties on the GSHP design process. A relative measure of the thermal energy depletion of the ground will be used to quantify the influence of the GSHP operation on temperature field in the ground under the uncertainty in thermal conductivity coefficient.

## 2. Evaluation of Geological Environment from the Perspective Projection BHE

Measurement and evaluation of key parameters of rock environment from the viewpoint of its applicability for various BHE installations is based not only on current knowledge of the rock environment, but also on knowledge of tools, materials and technologies of design, installation and their operation. Rock environment is described in case of application of a BHE in a GSHP system.

An appropriate design of BHE parameters is a prerequisite for its efficient and economical operation. The design must allow for the specific mode of operation of the BHE [25], take into account the physical parameters of the rock environment [26], the geometric and physical parameters of the structural elements of the BHE [27] and the existing temperature field at the sampling site [28,29]. The physical parameters of the rock environment that control heat transfer are the thermal conductivity coefficient (λ), specific heat capacity (C${}_{p}$) and density (ρ). These parameters define the thermal diffusivity (α) through the Equation (1). Under the influence of flowing groundwater, it is also appropriate to determine the permeability coefficient (*k*).

(1) $$\alpha =\frac{\lambda }{C_{p}\rho }.$$

The values are determined on the basis of information about the rock environment obtained from various archival sources. The most important source is the archive of the Czech Geological Survey that gathers results from all types of geological surveys carried out by state institutions as well as commercial entities. Key sources of information are final reports of geological surveys and specialized maps characterizing the rock environment.

From the point of view of methodical procedure, small installations up to 50 MW${}_{th}$, usually including a single borehole for home and water heating, are completely separated. In this case the methodical procedures can be defined as empirical, based on the tabular value of power per 1 m of the given heat exchanger.

The actual dimensioning needs to be based on the knowledge of the given type of rock environment. If the data are not available, it is possible to use tabular values for standardized thermal characteristics of the given environment (i.e., rock type, aquifer, etc.) and thus carry out optimization using an analytical model.

As for installations in complicated geological and hydrogeological conditions, it is advisable to create a three-dimensional (3D) model of the rock environment at the site of the planned borehole heat exchange installation. The BHE thus becomes the basis for further modelling and simulation of heat energy flows in the surrounding rock mass. An example of complicated geological conditions can be a BHE situated in tectonically damaged crystalline basement with intensive influence of flowing groundwater. Most often it is based on specialized maps documenting the rock environment at the site of the planned installation with the projection of BHE positions. It is also necessary to gather data from all available sources documenting the vertical profile of the rock massif to the required depth (approximately 150 to 200 m). These drilling profiles are annexed to final reports on geological surveys archived by national geological funds. In case of insufficient data, it is necessary to propose exploration and drilling work to determine the necessary data. Based on the obtained data it is possible to create a 3D model representing the geological conditions at the installation site.

As for larger installations–medium installations in the output range of 50 to 300 MW${}_{th}$ and large installations above 300 MW${}_{th}$–it is advisable to combine methods for thermal characteristic determination in situ (e.g., by using an optical cable) with methods of laboratory determination of thermal conductivity.

It is suitable to carry out the optimization of the BHE using numerical models (e.g., FEFLOW–WASY Berlin, alternatively COMSOL Multiphysics or EED) and optimization algorithms (e.g., .NET components by OPTIM). Although building a numerical model is very demanding as for input data, it allows simulation of heat transfer both by conduction and convection using complex boundary conditions in heterogeneous rock environment, which reflects the physical reality of the problem more accurately, compared to analytical approaches. This way it is possible to ensure trouble-free operation for more than the first decades, to optimize the design according to complex consumption requirements, or to provide sufficient spare capacity for heating.

In this case it is appropriate to analyse also other properties of the rock environment:

- basic rock mass macrostructure and stratification,
- petrographic characteristics of the rock material (all types of rock) in the active zone,
- water saturation in the open pore system of the rock material,
- hydrogeological characteristics of the rock massif, especially groundwater flow.

## 3. Utilised Research Infrastructure

### 3.1. Characteristics of Research Infrastructure

In the vicinity of VSB–Technical University of Ostrava, two research geothermal stations with boreholes configured in polygons are currently being used for applied research of temperature changes in massive rock, the so called the “Large Research Polygon” and “Small Research Polygon”.

The “Large Research Polygon” is predominantly used for observing changes in the subsurface thermal profile in an area under the influence of large scale withdrawals of heat from the bedrock, serving as the main heating source for the University assembly hall (9234 m${}^{2}$ of floor space with calculated heat loss 1.2 MW at the outside temperature −15 ${}^{\circ }$C).

There are 10 installed heat pumps (type Greenline D70) manufactured by IVT Industries with combined heating power of 700 kW at (B0/W50). The system includes 110 boreholes drilled to a depth of 140 m. This research focused on 10 operating boreholes and 5 monitoring boreholes. Analog PT1000 temperature sensors (Dallas type) are installed at the depths of 20, 50, 100 and 140 m.

**Figure 1:**
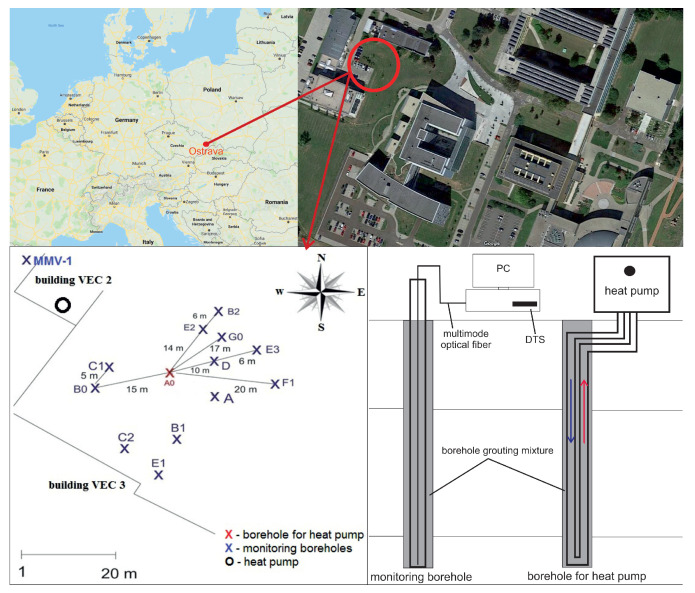
Location of boreholes in the *Small Research Polygon* geothermal station.

The “Small Research Polygon” (Figure 1) is dedicated to study specifically the regenerative and cumulative storage of heat in massive rockthe ground surrounding a BTES BHE designed to represent a small residential installation scale. This station is located at the building of Research Energy Centre (VEC) with one producing BHE (A0). This borehole is connected to two heat pumps manufactured by IVT Industries and located in the building VEC 2. The heat pumps are Greenline E 11 Plus with a heating power of 2 × 11 kW at B0/W50. The polygon array around the borehole A0 contains 11 monitoring boreholes (B0, B1, B2, C1, C2, D, E1, E2, E3, F1, G0). All boreholes (including A0) are drilled to a depth of 140 m. One of the monitoring boreholes (A) with the depth of 160 m is a part of the polygon. The measurable depths of the individual boreholes are shown in Table 2. Table 3 then shows the average surface air temperatures at the time of the measurement.

The array also includes 1 shallow monitoring borehole (MMV-1), drilled to a depth of 20 m. This borehole is used to monitor temperature variations in the upper part of the rock caused by seasonal changes in atmospheric temperature. The direction of the ground water flow was taken into account during the design of the research polygon and the choice of BHE positions. In order to measure the temperature changes in the rock, the DTS was used to take continual measurements along the whole length of the borehole.

### 3.2. Ground Characterisation from the Thermal Energy Production Point of View

In the very premises of the University, it is possible to locate a contact of Bohemian Massif and Western Carpathians. Based on the drill cuttings observation, accompanied by well logging, the detailed profile of rock environment in the vicinity of “Small Research Polygon” was established up to the depth of 160 m (see Table 4) [4].

The physical parameters of individual formations were estimated in a number of ways. A thermal response test (TRT) was performed on the monitoring BHE A providing us with an effective value of thermal conductivity of the strata. Rock samples produced during drilling of the BHE were analysed in laboratories of CAS (Czech Academy of Sciences). Several samples of the Quarternary rocks were analysed giving us a range for λ and volumetric heat capacity C${}_{V}$ values. The same laboratory analysed two samples of the Carboniferous Hradec Kyjovice formation. We used temperature logs in MMV-1 at 0 and 11 m of its length to estimate α of the quarternary formation by the periodic heat flow method [30,31]. We have used representative data published in research papers to complete the set of needed physical properties.

The published values of λ = 2.6 ± 0.71 W m${}^{-1}$ K${}^{-1}$ for lower Carboniferous sandstones and λ = 2.11 ± 0.34 W m${}^{-1}$ K${}^{-1}$ for lower Carboniferous siltstones [32] correspond very well to our laboratory values.

On that account we have used the published value of λ = 2.11 W m${}^{-1}$ K${}^{-1}$ for Miocene sediments. A published value of the heat flow density that is representative for the location of the research polygon is *q* = 71 mW m${}^{2}$ [33]. The heat flow density is defined by the Fourier’s law as Equation (2):

(2) $$q=-\lambda \frac{\partial T}{\partial z}.$$

The characteristics of the ground (especially petrographic, structurally geologic and hydrologic) precondition the utilization of its thermal energy. It is necessary to respect especially thermal conductivity of massive rock as a whole and, concerning the larger installations, also the thermal conductivity of individual minerals that is dependent on structural properties, type and mineral composition of rock as well as thermal capacitance of minerals. Minerals containing higher share of silica have the highest conductivity, while the lowest conductivity belongs to siltstone and claystone [4,34].

Production of the thermal energy from the ground is achieved by one BHE (A0). This BHE is 140 m deep and has a diameter of 120 mm. Its completion was done with double HDPE U-tube with 40 mm tube diameter. The remaining voids were grouted with a cement bentonite mixture with λ=0.6 W m${}^{-1}$ K${}^{-1}$. The Thermal Response Test was also conducted on the BHE A0 and its thermal resistance was evaluated at R${}_{b}$ = 0.17 mK W${}^{-1}$ [35]. The thermal resistance of the A0 BHE was also evaluated with EED software functionality as R${}_{b}^{EED}$ = 0.165 mK W${}^{-1}$. The two estimates correspond very well to each other. Nevertheless, performing a Thermal Response Test (TRT) is recommended for larger heat pumps installations with heat losses above 50 kW. The TRT measurement results in more accurate information on the thermal conductivity of the ground, the thermal resistance of the borehole and the unaffected ground temperature. Numerical and analytical modelling software programs are used to recalculate the required range of a larger set of BHEs in order to avoid thermal interference.

Yearly climatic conditions in the area are predominantly dependent on the variable amount of incoming sunlight. While the average intensity of sunlight hitting the outer border of Earth’s atmosphere is approximately 1360 W m${}^{-2}$, the actual intensity of radiation (energetic rays) reaching the Earth’s surface is lower.

The intensity of radiation impacting the Earth’s surface in the Czech Republic oscillates between 230 and 260 W m${}^{-2}$, which equates to a yearly irradiation of approximately 1000 to 1140 kWh m${}^{-2}$. Short -lived maximums of intensity may exceed 1000 W m${}^{-2}$ under exceptional conditions.

The total amount of sunlight in the Czech Republic ranges between 1000 to 1700 h per year (according to the location of place) during an average year and supplies corresponding amount of energy. The amount of sunlight is not equally distributed throughout the year. During the coldest half of the year (beginning in October and ending in March), only 25 % of the sunlight hits the earth, while during April to September the remaining 75 % is received.

### 3.3. Analysis of the Ground Temperature Field

**Figure 2:**
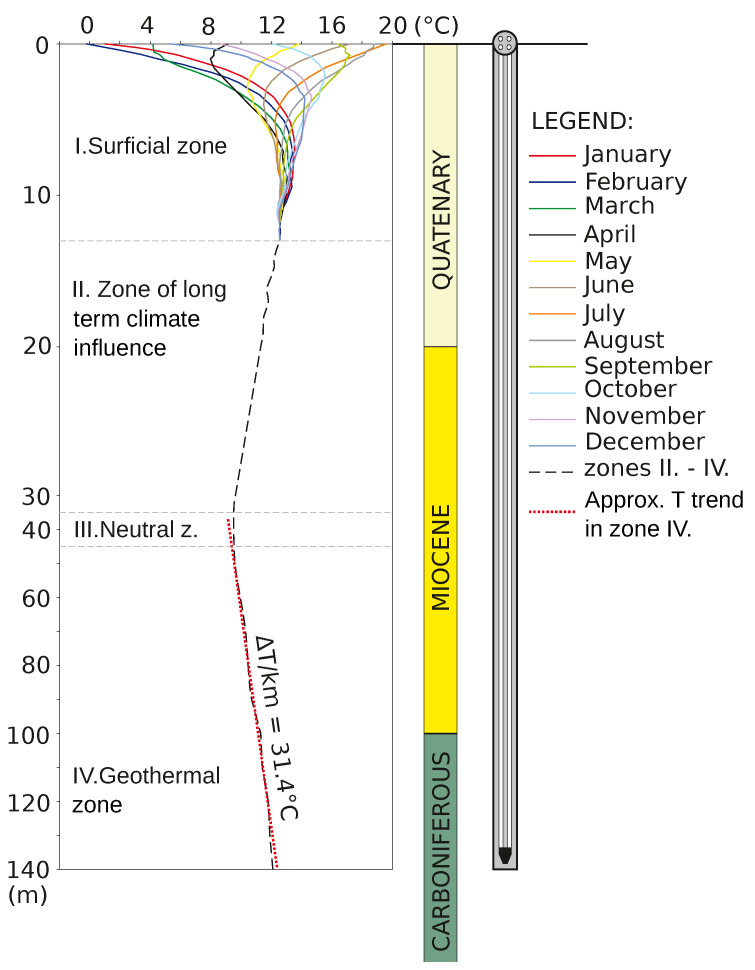
The structure of rock environment at Small Research Polygon according to the temperature influences of individual zones [35].

Based on the long-term observations at the Small Research Polygon, the subsurface can be divided into four zones (Figure 2):

- **Surficial zone**: At the BHE location, given the physical parameters of the ground and amplitude of surface temperature variations, this zone reaches a depth of approximately 12 m. In this interval, ground temperatures are affected by seasonal variations of temperature (and other climatic parameters) on the land surface. Longer duration (inertial) effects are detected in “winter” months. This zone has the largest heat potential during the summer, then, approximately in September, the heat flow reverses depending on climatic conditions to the ascending stage and passes on the heat energy. Due to the delay of temperature changes at the depth of 2 m and further, the heat flows reverse to ascending direction several months later, i.e., the end of autumn, the beginning of winter.
- **Neutral (transition) zone**: The temperature in this zone drops gradually to its lowest temperature at the bottom in the transition zone to zone3. The thickness of this zone is approximately 30 m. In deeper layers, the temperature fluctuations are dampened down to the transition zone. From the operational point of view, the heat-carrying medium in the ascending branch of collector has the highest temperatures in these surficial and shallow sectors. Considering this observation, the fact that heating is utilized especially during the period starting approximately in October, when this highest zone begins to pass the heat closer to the surface and reaches the lowest temperatures, the most significant heat loss occurs there out of all ascending branch.
- **Zone of long-term climate influence**: Designates the area below the “lowest” temperature which equals the value of long-term yearly average temperature on the long-term yearly average temperature on the surface. For the given location this temperature is 8 ${}^{\circ }$C, between a depth of 35–45 m.
- **“Geothermal” zone**: Expected rise of temperatures according to grade (i.e., gradient, occurs in this zone). For the Ostrava area, the thermal gradient is ΔT/km = 31.4 ${}^{\circ }$C. Going deeper, the temperature drops until the transition zone, from where the temperature increases with depth. The thermal profile was constant since the start of the well monitoring. This long-term influenced zone often constitutes the main location where the collector is installed. It typically a exhibits minimal supply of heat from the surface and the supply of heat from the deeper parts of the rock massive is prevalent here. The higher thermal conductivity of the rock, the higher heat flow can be expected.

## 4. Used Methods and Devices for Measurements and Temperature Logging of BHEs

As mentioned at the beginning of the article, we have used the DTS system to measure temperature changes of the rock mass through measured thermal boreholes in a polygon [36,37,38]. The technology of the DTS system is based on the principle of optical reflectometer, i.e., a light impulse, at wavelength of 975 nm, 1064 nm or 1550 nm according to the type of DTS and width of 10 ns, is released into the filament. A certain portion of the light impulse returns back to the DTS at the original wavelength (elastic–Rayleigh scattering) and at different wavelength (non-elastic scattering). Non-elastic phenomena, causing recurrence of the portion of the light impulse back to DTS system, are called Raman’s and Brillouin’s stimulated scattering. DTS systems are therefore distinguished according to the utilized type of scattering which they employ. DTS that detect Raman stimulated scattering use a multimode optical fibre (diameters of the core and the cladding are 50 μm and 125 μm, respectively) with high value of numerical aperture for maximization of the conducted intensity of the light reflected back because the volume of the reflected Raman’s stimulated scattering is relatively small. Relatively higher inhibiting characteristic of a multimode optical fibre limits the range of such DTS systems to 8–10 km. Contrary to Brillouin Time Domain Reflectometry (BOTDR), DTS detecting Brillouin’s stimulated scattering employs a single mode optical fibre (diameters of the core and the cladding are 9 μm and 125 μm respectively) and they are able to measure temperature and mechanical stress up to more than 30 km. Spatial resolution of DTS system is usually 1 m at thermal resolution 0.01 ${}^{\circ }$C. Extreme DTS systems (using Brillouin stimulated scattering) have spatial resolution 0.5 m and thermal resolution 0.05 ${}^{\circ }$C. They are particularly accurate and precise measuring systems [39].

If we want to determine temperature at a certain point of the optical fibre *z* (distance from the front of the optical fibre), it is necessary to focus first on the Raman scattering spectrum. I${}_{S}$ represents the intensity of the Stokes part of the Raman scattering, I${}_{AS}$ is the intensity of the anti-Stokes part of the Raman Scattering. Relations describing the intensity of the returning parts of the Raman scattering are described below (Equations (3) and (4)). The same equation for attenuation from the start to the point of the fibre z applies for both relations. However, commonly used lasers in optical-fibre DTS based on Raman stimulated scattering have a wavelength of 1064 nm. In this case, the peaks of parts of the Raman spectrum will be shifted ±40 nm, so the wavelength will be 1104 nm and 1024 nm. Since attenuation is a function of the wavelength, this phenomenon may cause an error in temperature determination by:

(3) $$I_{S}\left(z\right)=C_{S}\;e^{-\alpha_{R}\;z}\;e^{-\alpha_{S}\;z}\langle n_{k}\rangle ,$$

(4) $$I_{AS}\left(z\right)=C_{AS}\;e^{-\alpha_{R}\cdot z}\;e^{-\alpha_{AS}\;z}(\langle n_{k}\rangle +1),$$

where T(z) is the size of temperature at the spot *z* and $C_{S}$, $C_{AS}$ are constants, and $\langle n_{k}\rangle$, by Equation (5):

(5) $$\langle n_{k}\rangle =\frac{e^{-\frac{h\Omega }{kT(z)}}}{1-e^{-\frac{h\Omega }{kT(z)}}},$$

where *h* is the reduced Planck’s constant, *k* is the Boltzmann constant, (2πΩ) is the red and blue frequency shift. The most important part for fibre optic distributed systems using stimulated Raman scattering in optical fibres is the anti-Stokes part of the spectrum. This part of the spectrum changes intensity by changing the temperature along the optical fibre profile. The Stokes part of the spectrum shows temperature independence. That is why DTSs work on the basis of the ratio of the anti-Stokes part intensity to the Stokes part intensity [40] by Equation (6):

(6) $$\frac{I_{S}\left(z\right)}{I_{A}S\left(z\right)}=\frac{C_{S}}{C_{A}S}\;e^{-\Delta \alpha z}\;e^{-\frac{h\Omega }{kT(z)}},$$

where we can express $\Delta \alpha =\alpha_{S}-\alpha_{AS}$ and is greater than zero. The final equation describing the principle of operation of optical-fibre DTS based on stimulated Raman scattering is a linear combination of the temperature offset (the first part of Equation (7)), attenuation difference in optical fibre (the second part of Equation (7)) and measured temperature based on the ratio of anti-Stokes and Stokes parts of the Raman spectrum (the third part of Equation (7)):

(7) $$T\left(z\right)≅T_{REF}\left(1+\frac{\Delta \alpha z}{ln\frac{C_{S}}{C_{AS}}}+\frac{ln\left(\frac{I_{S}\left(z\right)}{I_{AS}\left(z\right)}\right)}{ln\frac{C_{S}}{C_{AS}}}\right),$$

where the T${}_{REF}$ reference temperature offset corresponds to Equation (8):

(8) $$T_{REF}=\frac{h\Omega }{k\;ln\frac{C_{S}}{C_{AS}}}.$$

However, it is always necessary to ensure that the DTS measuring system is correctly calibrated in combination with the type of optical cable used as the sensor tool before the actual measurement. There are a number of ways to calibrate a DTS device (manually, automatically), but all types of calibrations must be done very precisely in order to use the measured results [41,42].

### 4.1. Methodology of Measurements

The inquiry into the temperature changes of energetically utilized rock massive deals with the heat gained from the rocks at the upper part of the earth crust, whose main source is especially sunlight, as well as with the inner sources of the Earth (the heat released by the influence of tectonic and volcanic activity, radioactive decay of elements, etc.) [36]. Operation of a heat pump connected to a collector situated in a borehole disturbs the thermal equilibrium in the surroundings that leads to the transmission of heat inside the rock towards the borehole. The transmission of heat is facilitated by the fluctuation of crystalline structured of minerals (so-called conduction) that constitute the rock, which can be designated as dry heat contrary to the heat transferred by the flow of ground water (convection) [7,43]. In our case, at the beginning of the measurement of the temperature changes of energetically utilized ground around each measured boreholes, we conducted an analysis of the quality of the used multimode fibre optic cable OM2 (with typical parameter of optical fiber was 50/125 μm) using the reflectometric method OTDR, where we monitored whether the label is damaged and there is no change in attenuation or given types of losses somewhere along its entire length. If there is an increased occurrence of attenuation along the fibre optic cable, it would be a consequence of the measurement itself or the evaluation of changes in temperature events by the DTS system. After measuring the cable with OTDR, we have recommended by Sensornet Industries a manual procedure for manually calibrating the instrument to a particular type of sensory multimode fibre cable used. Before the start of each borehole measurement, an additional calibrated mercury thermometer was used to validate the temperature values along the optical fibre measured by the DTS system. Each of the measured geothermal boreholes is equipped with a PE tube with a diameter of 30 mm. An optical cable with appropriate protection against damage was then inserted into these PE tubes and after a certain time of installation the measurements were performed.

On the recording day, a measuring multi-mode fibre was inserted into every chosen monitoring borehole where it was continually measuring temperature changes of the rock massive. Individual measurements lasted approximately 5 min for a single observed borehole. Measured values were saved by the driving unit of the DTS system, where the data was consequently evaluated.

### 4.2. Energetically Utilized Operational Borehole A0

The EED software was used to design BHE A0. Input parameters for the design were either measured (thermal conductivity of the grout mixture and HDPE U-tube) or estimated (heat flow density and λ from literature, undisturbed ground temperature as a yearly mean air temperature). BHE was designed to cover a heat demand of 20 MWh during one heating season. BHE was optimised as a single 140 m long borehole equipped with double U-tube. Utility of EED software estimated the thermal resistance of the BHE A0 as R${}_{b}^{EED}$ = 0.165 mKW${}^{-1}$. A thermal response test later evaluated its resistance as R${}_{b}^{TRT}$ = 0.171 mKW${}^{-1}$. During the heating season (September–May), 16,499.7 kWh of heat was produced with total energy consumption of 6068.6 kWh (where 6006.0 corresponds to the heat production time). The difference of these values expresses the consumption of heat pumps in standby mode. Seasonal heating factor related to the total consumption of electricity is 2.72. Total physical withdrawal from the operational borehole A0 was 11,096.3 kWh, while 10,493.7 kWh was used effectively.

**Figure 3:**
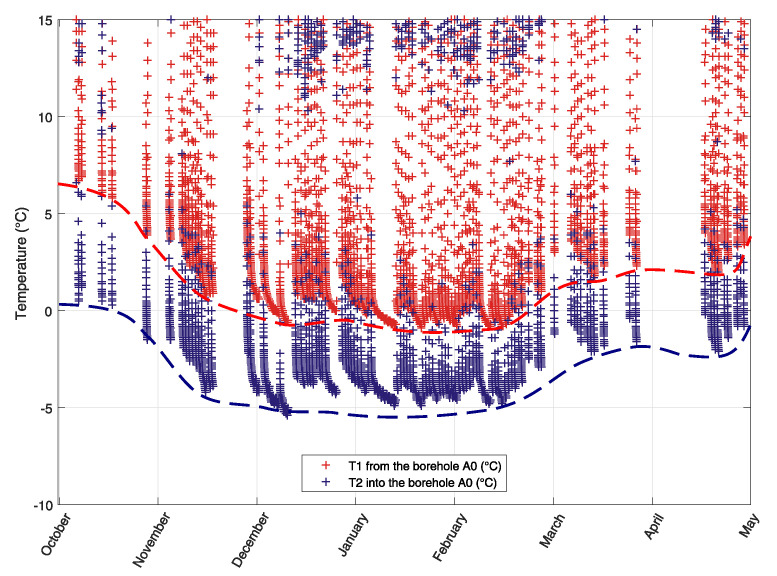
Development of temperature changes in energetically utilized operational borehole A0 during one heating season.

Figure 3 shows the temperatures of brine (mixture of water and ethanol) flowing from (from-red) to (into-blue) recessed collectors in borehole A0.

Inlet and outlet temperatures of brine were measured in a section of the circulation pipes located in the technical room, which means that during longer heat pump shutdowns, the brine temperatures were equalized with the room air temperature, which was about 20 ${}^{\circ }$C. When the heat pump was started, the temperatures were then rapidly lowered to a level where both brine temperatures were no longer affected by the temperature in the utility room. In the graph these states are always visible in the lower part of each course, where the temperature drop is already slower and indicates a gradual cooling of massive rock (points representing these states are more inflated).

**Figure 4:**
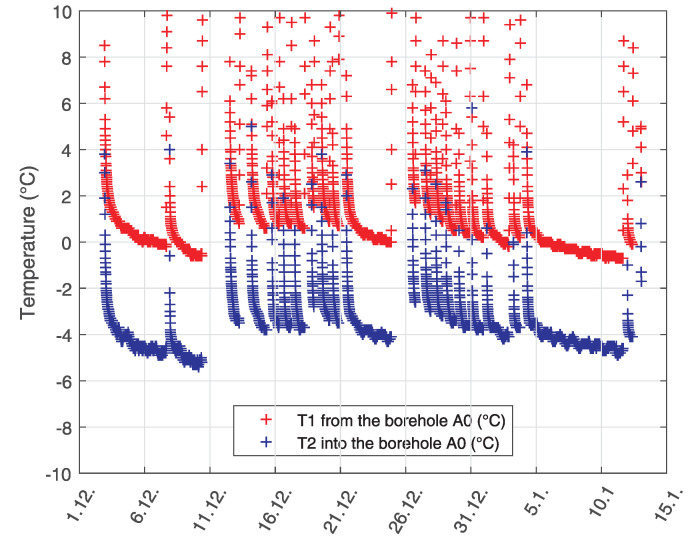
Time detail of the temperature change course in the A0 borehole with the lowest temperatures.

The longest period of continuous operation was from 5 January to 13 January. This section can be seen approximately in the middle of the time axis in the graph of Figure 3, and at the end of the time axis in the graph of Figure 4 which shows time detail of temperature changes with the lowest temperatures. It can be seen from the graphs that the limit temperatures of the liquid in the borehole drop at the end of the calendar year, after which they are approximately the same by the end of February, followed by an increase in temperature. This is a sign of a well-designed borehole when there is no permanent drop in temperature during the heating period, but it can be seen that the borehole is already being regenerated after the end of the main heating season Figure 3.

## 5. Results of Measuring Temperature Changes in Energetically Used Rock Massive

**Figure 5:**
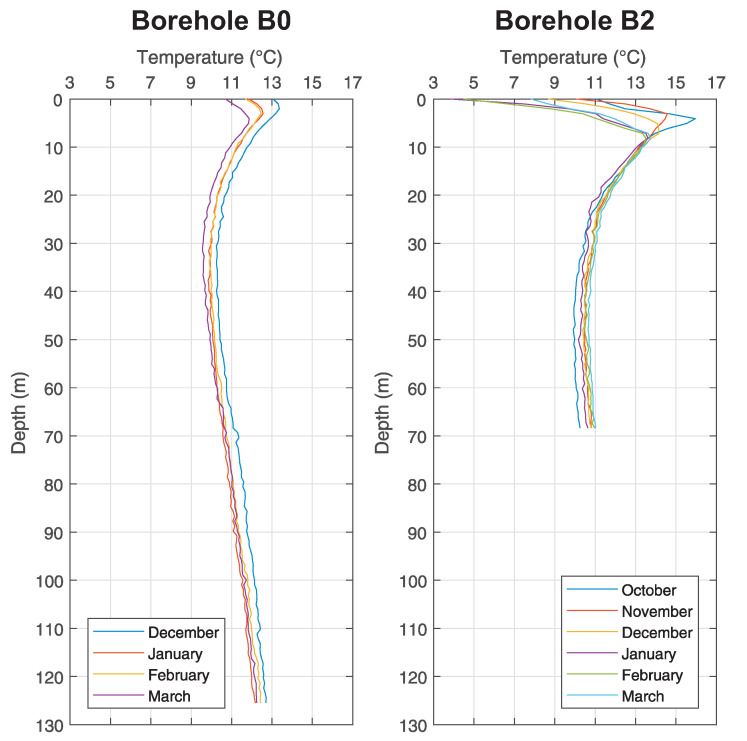
Graph of temperature changes with the depth at chosen monitoring borehole B0 and B2 of the *Small Research Polygon*.

**Figure 6:**
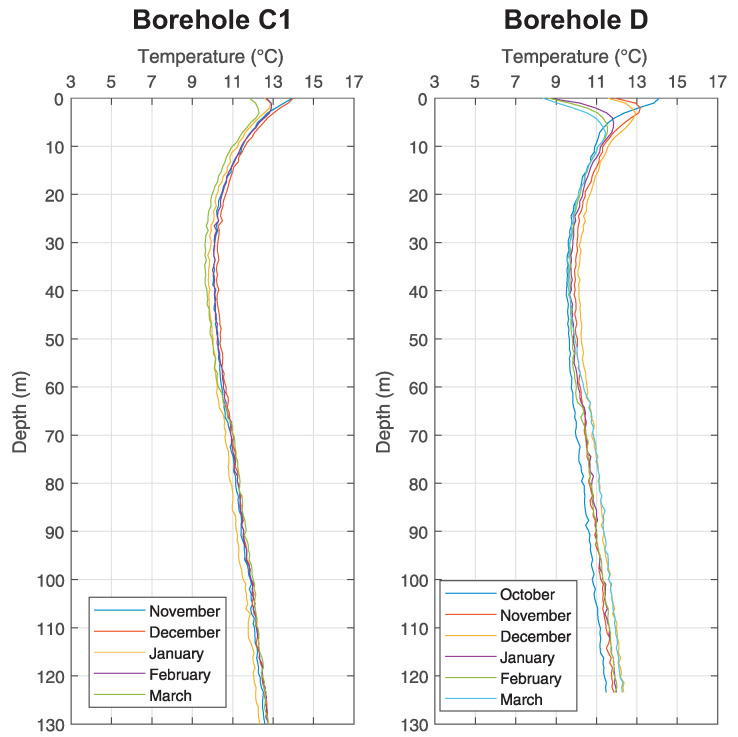
Graph of temperature changes with the depth at chosen monitoring borehole C1 and D of the *Small Research Polygon*.

**Figure 7:**
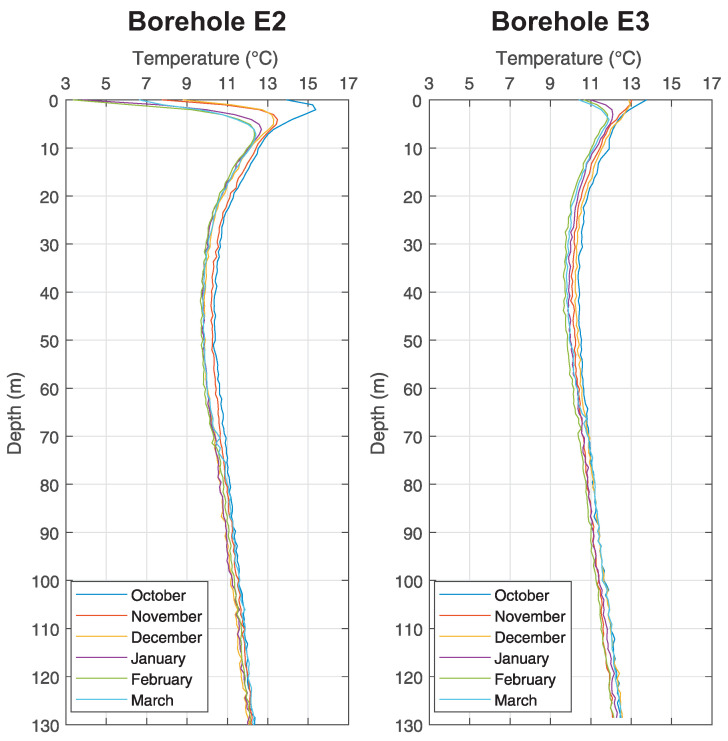
Graph of temperature changes with the depth at chosen monitoring borehole E2 and E3 of the *Small Research Polygon*.

**Figure 8:**
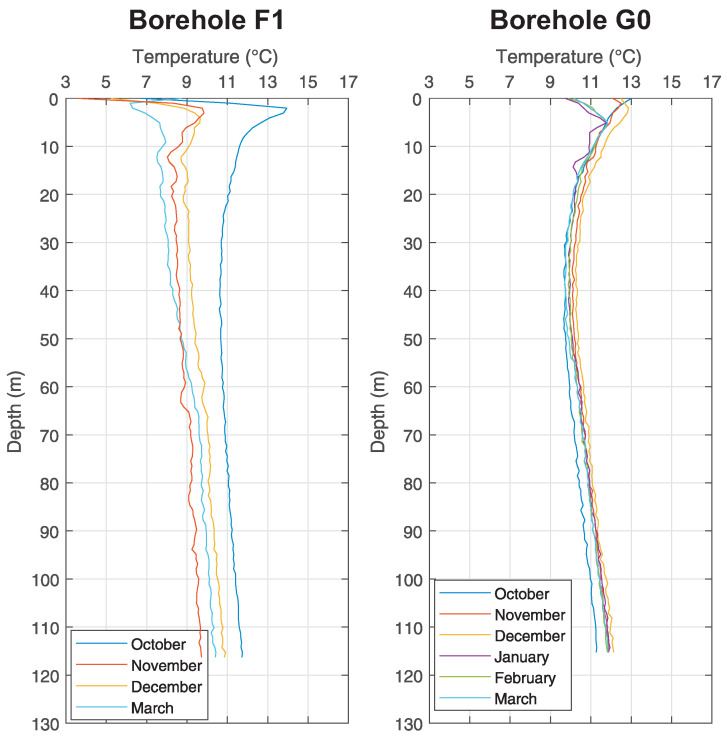
Graph of temperature changes with the depth at chosen monitoring borehole F1 and G0 of the *Small Research Polygon*.

The measurements by means of fibre optics took place in the middle of each month from October 2016 until March 2017 at the *Small Research Polygon* station. All boreholes were drilled up to 140 m, but transits for measurements were at various depths (see Table 2). Figure 5 shows boreholes B0 and B2, Figure 6 shows boreholes C1 and D, Figure 7 shows boreholes E2 and E3, Figure 8 shows boreholes F1 and G0, shows final developments of temperatures of the rock massive in a given month of measuring. Introductory temperature values at interval 1–2 m are influenced by the placement of monitoring boreholes into the chambers (impact of measurements by surface temperatures) which are located slightly under the terrain level.

### 5.1. Temperature Log Analysis

In case of analysis it is possible to infer from these results that the temperatures of the ground in a close distance around the BHE (up to 20 m) are not influenced by its operation at all, as seen in Figure 5, Figure 6, Figure 7 and Figure 8.

As discovered by measuring inside the monitoring boreholes, there is constant temperature between 11 ${}^{\circ }$C and 12 ${}^{\circ }$C at the depth about 100 m, despite any changes in surface temperatures and despite the operation of BHE. Waveforms are constant inside all monitoring boreholes, regardless of their distance from BHE A0 (see Figure 5). Small variations, visible in graphs, are caused by weak flow of ground water and by possible damage of grouting mixture around the HDPE tubes of monitoring boreholes (especially in the case of monitoring borehole F1 (see Figure 8) it is possible to interpret variations of individual waveforms as inappropriately grouted space between U-tubes and the wall of the borehole).

The analysis of the temperature logs identified the region of quasistable heat flow below the transition zone in the ground. We have therefore used the data to evaluate the temperature gradient with depth in this geothermal zone. It allowed us to estimate the effective thermal conductivity (λ) of the ground (see Table 5) as the representative heat flow density (q) in our region is known as 70.7 m W m${}^{-2}$ with confidence limit given by a standard deviation of 3.3 m W m${}^{-2}$ [33], which makes a relative error of *q*≈ 4.7%. We have used measured temperatures from depths larger than 60 m down to the BHE’s measurable depth to calculate a temperature gradient in a borehole.

Subsequently, we have utilised the value of *q* to estimate an effective coefficient of thermal conductivity (λ) of the ground using the Fourier’s Equation (9):

(9) $$q=-\lambda \cdot grad\left(T\right).$$

The relative error propagating through this expression can be estimated using multiplication rules on Equation (10):

(10) $$q\pm q_{err}=-\left(\lambda \pm \lambda_{err}\right)\cdot \left(grad\left(T\right)\pm grad{\left(T\right)}_{err}\right)$$

resulting after few assumptions and simplifications into Equation (11):

(11) $$\frac{q_{err}}{q}=\frac{\lambda_{err}}{\lambda }+\frac{grad{\left(T\right)}_{err}}{grad\left(T\right)}$$

We’ve evaluated the error of $T_{err}$ as a standard deviation of residuals of the linear fit that was used for a grad(T) estimation. Then we calculated an error of the temperature gradient as a difference of the observed one and one influenced by temperatures with $T_{err}$ included. We’ve then used the values to evaluate the relative error of λ. We’ve included estimated relative errors along the calculated values of λ as can be seen in the Table 5.

The results showed unexpected variation in estimated thermal conductivities not only among individual boreholes but with each measurement in any individual borehole as well. We can see that the estimated thermal conductivities are reasonably close to the one obtained with thermal response test on BHE A0 (2.4 mKW${}^{-1}$). Spatial variation of λ was expected. However, the temporal variation of λ was surprising. We have declined the likeliness of such an influence being caused by our measurement and research activities on the BHE field after a careful double check of measurement system calibration and the state of boreholes. The reason is most likely the long-term evolution of atmospheric conditions subtly influencing the vertical shape of temperature field even through depths larger than 90 m and between time steps of about 1 month.

### 5.2. Analysis of Design Input Parameters Influence on BHE Performance

**Figure 9:**
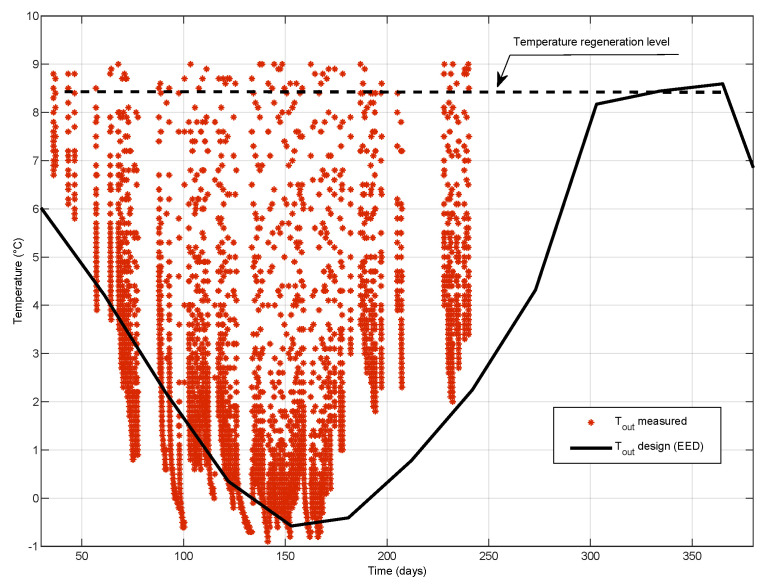
Temperatures of the fluid at the outlet of BHE A0 as predicted with EED software during the design (solid line) and measured during production (red dot).

We have presented the performance of the BHE A0 during its operation for one heating season. We have compared the measured temperatures at the outlet of the BHE (T${}_{out}$) with their predictions made by the software EED (see Figure 9). We can see that there is a certain level of mismatch. The designed thermal energy demand is more evenly distributed throughout the heating season than the real one. The real energy production was not continuous and consists of many intervals of more intensive depletion than the design expected. However, the total heat production was reasonably corresponding to the design.

We have made an attempt to quantify the influence of the uncertainty of the input parameter on the BHE design. We have simulated several scenarios in EED software in the following manner. We have kept most of the simulation inputs constant (base heat load, BHE geometry–except its length, heat carrier fluid properties and double U-tube type of completion). We have solved the problem for the length of a single BHE with minimal and maximal (reasonable–see Table 5) values of λ, i.e., 2.24 and 2.96 W m${}^{-1}$ K${}^{-1}$ respectively. The optimal BHE lengths for these two scenarios were 142 m and 129 m respectively. We can see that an uncertainty in the ground thermal properties can lead to almost 10% differences in design of BHE length for this particular exercise. Another exercise was meant to present the thermal field disturbance by a producing BHE in the ground with uncertain thermal conductivities. The produced thermal energy (*W*) by a BHE can be obtained with known properties of heat carrying fluid (density ρ, heat capacity C${}_{p}$), its volumetric flow (*Q*) and thermal difference of the inlet and outlet temperatures (ΔT) over a time period (*t*) as Equation (12):

(12) $$W=Q\;\rho \;C_{p}\;\Delta T\;t.$$

**Figure 10:**
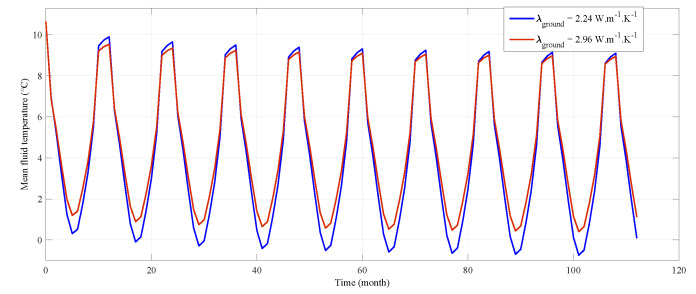
Mean of the fluid temperatures at the inlet and outlet of the BHE at the end of each month during the simulation period of 10 years. Each curve is representing a simulation at certain thermal conductivity of the ground while the other simulation parameters remain constant.

We have used the mean fluid temperatures ($\bar{T}$) calculated by EED software (see Figure 10) for the end of each month to get an estimate of a certain heat load effect on the thermal field in the ground. As in the previous example, where we dealt with the BHE length, we have used a smaller and a larger estimate of thermal conductivity for the ground properties in each simulation. The effect of the heat load on the ground can be quantified with integral form of Equation (13) as:

(13) $$\Delta W=\overset{=K}{\overset{︷}{Q\rho C_{p}}}\int_{0}^{\tau }{\bar{T}}_{\lambda \max}\left(t\right)-{\bar{T}}_{\lambda \min}\left(t\right)dt,$$

where $\lambda_{max}$ and $\lambda_{min}$ denotes the simulated cases with larger and smaller thermal conductivity, and τ is the length of the simulation period (10 years). It can be seen as a relative measure of the disturbance of the thermal field in the ground under the thermal energy production. We neglect the possible differences in ambient thermal fields in the ground at one geographical location because of slightly different values of λ. The heat load, the BHE length and its R${}_{b}$ were held constant in these two cases. We have obtained the result of ΔW = K40 kWh by integrating the $\bar{T}$ curves (Figure 10) as in Equation (13). The estimated mean temperature of the fluid at the end of A 50-year-long period of production in these two cases are 0.1 ${}^{\circ }$C for higher λ and −1.4 ${}^{\circ }$C for a case of smaller of the two λ. The ground is being depleted more intensely in a case of smaller ground thermal conductivity but with the radius of temperature drop around the BHE affecting smaller ground volume. Predicted recovery of the ground temperature is quicker in the case of smaller λ as the ambient temperatures are undisturbed relatively close to the BHE compared with wider (however less intensive) influence on the ground with larger λ. Both cases perform well for prescribed small heat load.

**Figure 11:**
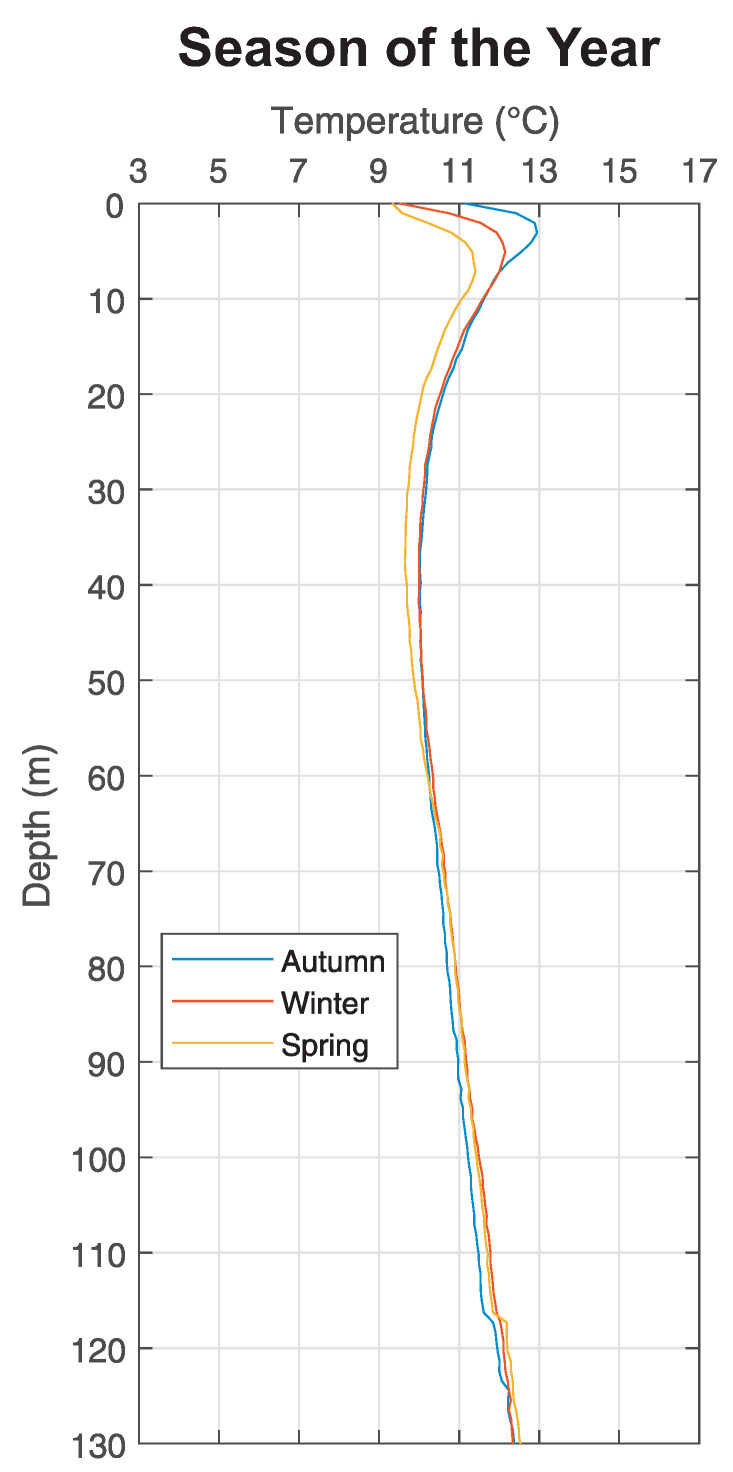
Average of measurements in boreholes in relation to seasons.

As a significant point of interest, we can observe that in the depth of approximately 38 m can be found a neutral zone–break point (shown in detail in (Figure 11), where the rock massive ceases to be influenced by long-term conditions at the surface and below this zone the temperature is increasing in accordance with thermal gradient.

**Figure 12:**
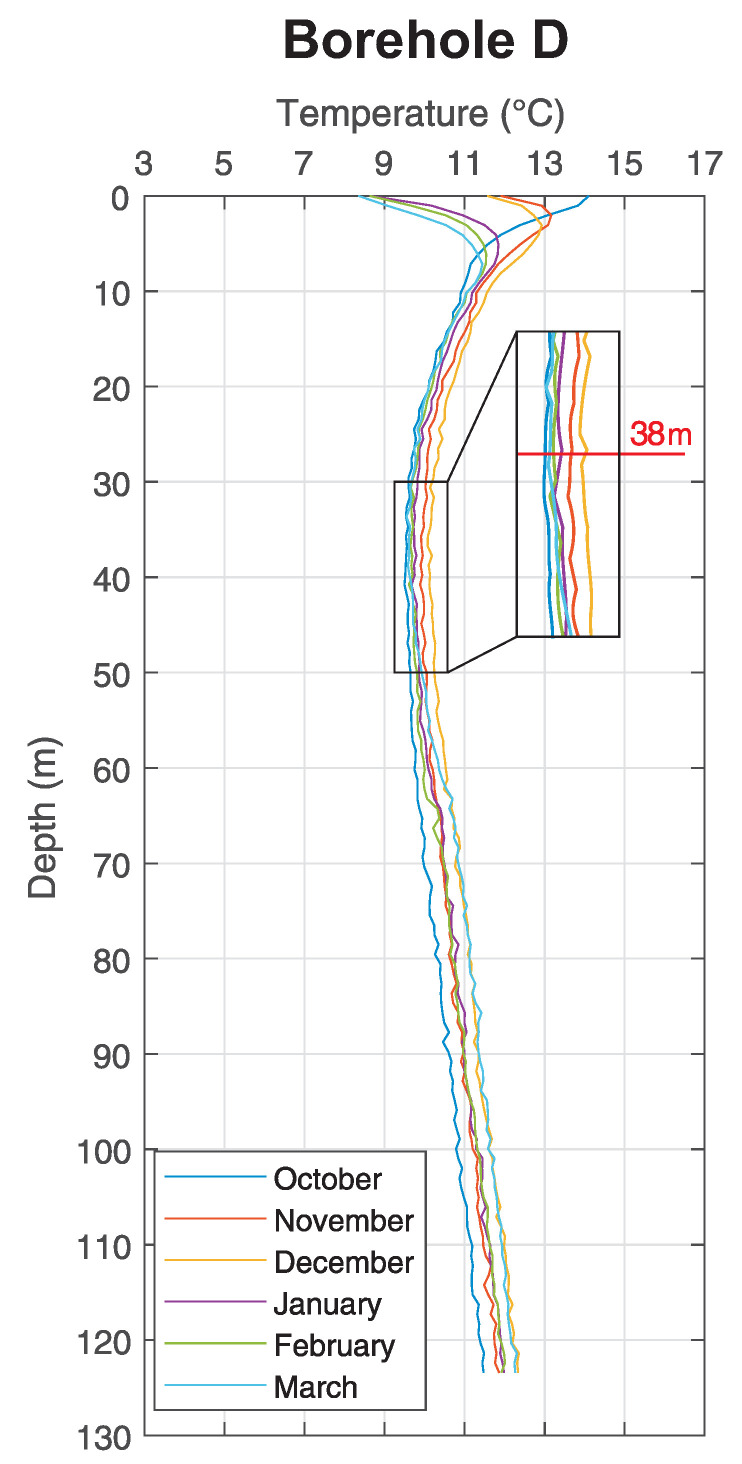
Neutral zone in the depth of approx. 38 m.

On the basis of our performed measurements during the whole year (Figure 12), we can observe that the operation of borehole heat exchangers for heating small buildings does not significantly influence the temperature the surrounding rock mass. Figure 12. shows averaged out measurements realized in all relevant boreholes (B0, B2, C1, D, E2, E3, F1 a G0) for various seasons (autumn, winter, spring). A more significant deviations of individual waveforms, visible up to the depth approx. 10–12 m, are caused predominantly by the influence of seasonal climatic changes on the surface in the so-called surficial zone. This zone is located from the entrance of borehole till the depth of approx. 40 m, where we can see that the temperature of the ground closer to the surface was highest in autumn and it was decreasing slightly in the following seasons. Even though the design of the BHE wasn’t based on measured data the system works reasonably well within our expectations.

## 6. Discussion

The analysis of the temperature logs and vertical zonation of the thermal field showed a few things. The long-term evolution of atmospheric temperatures influences the shape of temperature dependence on depth through the whole BHE length under the surficial zone. Such a convex function of depth in dry ground conditions can be explained only as a result of the end of the last little ice age in our region. It took place around 15 and 17 century and the surface temperatures kept reaching a climatic optimum since then [44]. The ideal state of a lower temperature near the surface was turned over due to the delaying and retarding effects of thermal diffusivity of the ground.

A similar effect was observed in Poland in a former permafrost [45]. The neutral zone therefore shifts according to relative atmospheric disturbances during decades. These changes in thermal field are slow in the geothermal zone, so we can consider it to be in a quasistatic state of heat flow. A temperature log through this zone of linear rise of temperatures can be used to estimate the thermal conductivity of the formation with Fourier’s Law (Equation (2)) [46].

Geothermal potential for heat pump systems is mostly based on evaluation of the coefficient thermal conductivity [47]. Thermal conductivity of the ground (rock) can be evaluated with laboratory measurements of the samples collected on outcrops and form a basis for thermal conductivity maps [48] or directly on core specimens directly sampled during the BHE drilling. However, the in situ thermal conductivity of the rock and soil materials is strongly dependent on water saturation and other pore geometry related parameters [49].

We have presented the attempt to evaluate the effective thermal conductivity of the ground around a certain segment of the length of BHE using the Fourier’s Law. This method was mentioned earlier in [46] in their paper and is usually adopted in regional heat flow density estimation [50,51]. Our measurements were based on the assumption that the temperature field is undisturbed in depths larger than 70 m in our particular setting. We have presented the case for a disturbance of the temperature field by a delayed response to the long-term evolution of atmospheric conditions.

A direct dynamic BHE testing by TRT method gives the most representative effective thermal conductivity coefficient values to the ground at the point of need at natural conditions. The experimental setting can, however, bring up a challenging task to include hydrogeological and hydrological studies to the table. We have personally encountered such a case on the Western margin of the Jeseníky mountains, a region strongly influenced by tectonics, during the spring thaw season. The TRT that we have conducted there evaluated the λ = 18 W m${}^{-1}$ K${}^{-1}$ [35]. As reported earlier was in [52], intensive groundwater flow can result in estimation of λ values up to 25 W m${}^{-1}$ K${}^{-1}$. Seasonal stability of the groundwater flow becomes a factor of the utmost importance in such instances. We have presented the way of obtaining λ for ground coupled heat pump design by several ways. We have used laboratory measurements on core samples, worked with relevant values published in literature, estimated λ with the Fourier’s Law and performed a Thermal Response Test on completed BHE. Thermal Response Tests provide the best hands on evaluation of the ground around the BHE and thermal resistance of the BHE itself. Using the measured values for different installations is, however, limited by a unique sequence of ground properties that contribute to the final effective value of λ.

Our analysis of the BHE production during one heating season showed that the system (see above) designed on predictions and best-informed assumptions available performs accordingly to the expectations. Our analysis of the best and the worst-case scenarios in terms of λ estimation suggests that the system with a small installation scale is not exceptionally sensitive to exact precision of input parameters for the system design. The temperature field around the BHE regenerates better without interference inside the field of multiple BHE involved. Our analysis of the relative depletion of the ground with Equation (4) brings the possibility to quantify the intensity of the ground thermal energy depletion. Shallow geothermal potential was estimated earlier as the intensity of the heat load on the ground with e.g., G.POT method developed in [53]. The operation mode was represented by the thermal load and its regime that can be either in storage or production modes. This approach can give us the ground for defining effectivity criteria upon our installation design by means of the critical fluid or ground temperatures [54].

As the operation mode influences the effectivity of a GCHP programme [25] and it is the building and its usage scheme that define the necessary heat load [55], its necessary to perceive the problem as an interplay of under and above ground components. We have made the case for the small installation being insensitive to variations of operational and ground parameters. Large installations with multiple interfering BHEs are in the focus of our future research.

## 7. Conclusions

From the results presented, we can therefore conclude that the operation of borehole heat exchangers for heating small buildings does not significantly influence the temperature the surrounding rock mass. Small scale installations seem to have a margin in design, where educated estimates of input parameters can lead to satisfactory operational results. Large installation with highly optimised operation in design can be more sensitive to certain deviations of production from prediction and face the limits of ground thermal energy availability.

The aim of this work was to prove how much the temperature changes during the season can manifest themselves on the behaviour of the borehole and the temperature gradient during the time of the borehole operation. At the same time, the aim was to point out that optimally dimensioned and drilled heat pump boreholes have a major impact on energy consumption or energy savings when confronting the use of conventional building energy sources. We can see that the parameters affecting the design of the BHE have clearly an important influence. Even though the possibility of obtaining the coefficient of thermal conductivity from temperature logs is appealing, it can lead to an error. Temperature field is being influenced by long-term atmospheric variations up to the depths of a conventional BHE. Deeper temperature logs are therefore necessary as the heat flow density at BHE’s vertical scale is not in a steady state.

The research has shown (see measurement on borehole F1) that the grouting mixture has a demonstrable negative effect on the “pumping” of heat from massive rock in terms of heat balance. This study can also fundamentally help in sizing depth and number of heat pump boreholes for smaller family houses. It is mainly the speed of influencing the rock mass during the heating season and its regeneration. It turns out that a smaller installation (1–2 wells for heat pumps in combination with a well-dimensioned heat pump) has no effect on the ground heat balance in terms of its operation and hence the optimization of well depths for heat pumps can be considered. Knowing the massive rock behaviour in terms of heat balance also opens the way to “storing” excess heat, for example from air conditioning.

Our simulations of heat production on a small-scale installation, done with EED software, showed that an uncertainty in design parameters has an influence on the thermal field of the ground. Relative measure of this influence showed that we can expect more intensive depletion of thermal energy of the ground if we overestimate its thermal conductivity. Our analysis shows that small-scale installations tend to have a certain tolerance to such deviations from the design.

## Figures and Tables

**Table 1:** Classification of GSHP systems according to the size of their installation.

Heat Pumps Installations	Year Consumption of Heat Energy (MW${}_{th}$)
Residential Houses	<50
Light Commercial	50–300
Commercial	>300

**Table 2:** Basic information about monitoring boreholes of *Small Research Polygon*.

Borehole	B0	B2	C1	D	E2	E3	F1	G0
Distance from the borehole A0 (m)	15	20	13	10	14	16	20	17
Measurable depth of boreholes (m)	125	68	130	123	130	128	116	115

**Table 3:** Surface air temperature at the time of measuring.

Month	October	November	December	January	February	March
Temperature (${}^{\circ }$C)	6.0	2.0	5.0	−3.5	1.0	6.0

**Table 4:** Detailed geological profile of *Small Research Polygon* as per borehole A0 [4], with representative physical parameters of the strata.

	Depth (m)	Type of Rock	$\lambda_{\mathrm{LAB}}$ (W m${}^{-1}$K${}^{-1}$)	$C_{V}$ (kJm${}^{-3}$K${}^{-1}$)	α (m${}^{2}$s${}^{-1}$)	$\lambda_{\mathrm{TRT}}$ (W m${}^{-1}$K${}^{-1}$)
Quar- ternary	0.0–2.5	Anthropogenic backfill	1.31–2.43	2.1–2.3	$7.56\times 10^{-7}$	2.4
	2.5–6.0	Claystone				
	6.0–7.0	Sandstone				
	7.0–8.0	Clayey sandstone				
	8.0–16.0	Claystone				
Miocene	16.0–22.0	Sandstone	1.88	-		
	22.0–29.0	Sandy claystone				
	29.0–113.0	Claystone				
Carbo- niferous	113.0–126.0	Siltstone	Siltstone: 1.85 Sandstone: 2.59	Siltstone: 1.71 Sandstone: 1.91		
	126.0–128.0	Silty sandstone				
	128.0–130.0	Siltstone				
	130.0–131.0	Silty sandstone				
	131.0–137.0	Siltstone				
	137.0–141.0	Silty sandstone				
	141.0–160.0	Siltstone				

**Table 5:** Effective thermal conductivity of the ground below 60 m depth for each borehole in each month of the measurement obtained with Equation (2). The mean and the standard deviation of the values in each borehole are included. (${}^{*}$ Less than 10 m of log was used, ${}^{**}$ Damaged grouting).

Borehole	λ ( W m${}^{-1}$ K${}^{-1}$)						Average Relative Error (%)
	Oct.	Nov.	Dec.	Jan.	Feb.	Mar.	
B0	-	-	2.48	2.50	2.28	2.45	8.9
* B2	4.20	2.52	5.59	2.88	3.03	3.67	70.8
C1	-	2.36	2.37	2.40	2.25	2.27	8.9
D	2.62	2.75	2.53	2.54	2.34	2.61	4.9
E2	2.96	2.69	2.53	2.37	2.28	2.24	4.8
E3	2.58	2.83	2.42	2.43	2.48	2.41	2.6
** F1	4.14	5.08	3.90	-	-	3.98	16.1
G0	2.78	2.66	2.56	2.48	2.57	2.57	1.9

## Data Availability

The data presented in this study are available on request from the corresponding author.

## Abbreviations

The following abbreviations are used in this manuscript:

**Symbol**	**Name**	**Unit**
*q*	Heat flow density	W· m${}^{-2}$
*t*	Time	s
$C_{p}$	Specific heat capacity	J·(kg·K)${}^{-1}$
$C_{v}$	Volumetric heat capacity	J·(m${}^{3}$·K)${}^{-1}$
*h*	reduced Planck’s constant	J/s 6.62607015·10${}^{-34}$
$I_{S}$	intensity of the Stokes part of the Raman scattering	
$I_{AS}$	intensity of the Stokes part of the Raman scattering	
*k*	Boltzmann constant	1.38064852·10${}^{-23}$ m${}^{2}$kgs${}^{-2}$K${}^{-1}$
*Q*	Volumetric flow rate	m${}^{3}$·s${}^{-1}$
*T*	Temperature	${}^{\circ }$C or K
T${}_{REF}$	reference temperature offset	${}^{\circ }$C or K
*W*	Thermal energy	W
α	Thermal diffusivity	m${}^{2}$·s${}^{-1}$
λ	Thermal conductivity	W·(m·K)${}^{-1}$
ρ	Density	kg·m${}^{3}$
*z*	distance from the front of the optical fibre	m

**Acronym**	**Term**
BHE	Borehole heat exchanger
BOTDR	Brillouin Time Domain Reflectometry
BTES	Borehole thermal energy storage
DTS	Distributed temperature system
EED	Earth Energy Designer (3rd party proprietary software)
GCHP	Ground coupled heat pump
GSHP	Ground source heat pump
HDPE	High Density Poly Ethylene
OTDR	Optical frequency time domain reflectometry
PE	Polyethylene
SCOP	Seasonal coefficient of performance
TRT (aka GeRT)	Thermal response test
